# 432. High-fat diet changed Salmonella infection patten associated with increasing intestinal permeability and altering gut microbiota

**DOI:** 10.1093/ofid/ofae631.146

**Published:** 2025-01-29

**Authors:** Xu Zhao, Chen lei, Xiaohan Guo

**Affiliations:** Institute of Huashan hospital, Fudan university, Shanghai, Shanghai, China (People's Republic); Shanghai General Hospital, Shanghai, Shanghai, China; Eye and ENT hospital of Fudan university, Shanghai, Shanghai, China

## Abstract

**Background:**

The impact of a High-fat diet (HFD) on gut microbiota may influence host susceptibility to pathogen infection. Here we utilized a HFD-induced dysbiosis mice model to investigate how HFD alters gut microbiota, thereby affecting the pattern of Salmonella infection.

**Figure:**
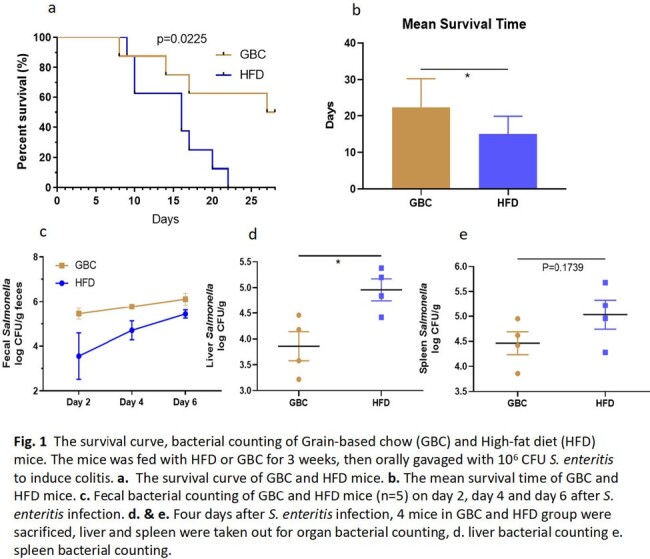

**Methods:**

The mice was fed with HFD or Grain-based chow (GBC) for 3 weeks, then orally gavaged with 10^6^ CFU *S. enteritis* to induce colitis. The survival rate, fecal bacterial counting, liver and spleen bacterial burden and cytokine detection were performed to compare the difference of two groups. Moreover, FTIC-Dextran method was used to investigate the intestinal permeability of HFD or GBC feeding mice. Finally, antibiotics cocktail pretreatment was performed to investigate the effect of HFD on gut microbiota.

**Figure:**
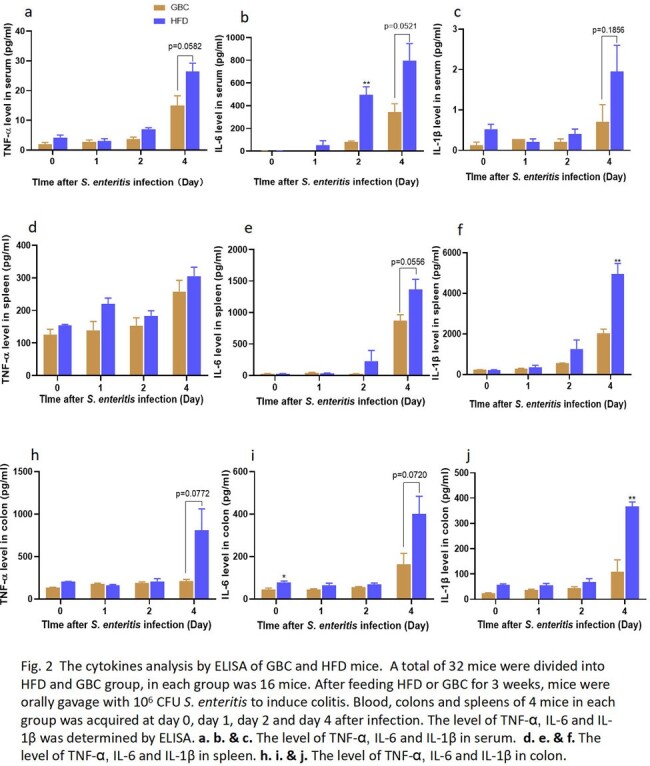

**Results:**

When the mice were performed gavage with 10^6^ CFU *S. enteritis*, the mortality of HFD mice (100%) was significantly higher than GBC mice (50%) (P=0.0225). Interestingly, the results displayed the fecal colonization amount of mice in GBC group was higher than HFD group. However, the bacterial burden of liver and spleen in HFD group was significantly higher than in GBC group (Fig. 1). The result showed the pro-imflammatory cytokine (TNF-α, IL-6 and IL-1β) concentration of HFD was higher than GBC mice, regardless of serum, spleen and colon (Fig. 2). Using FTIC-Dextran method to investigate the intestinal permeability, the results showed HFD decreased intestinal mucosal permeability. Meanwhile, Western results showed HFD reduced intestinal Occludin expression, which is an important component of the intestinal tight junction (Fig. 3). Finally, Antibiotics cocktail pretreatment was performed to destroy mice gut microbiota, the results showed the survival curve and fecal bacerial counting of group HFD+Antibiotics and GBC+Antibiotics was identical. It is implied that depleting gut microbiota with antibiotics rendered the mice equally susceptible to Salmonella infection, irrespective of their diets (Fig. 4).

**Figure:**
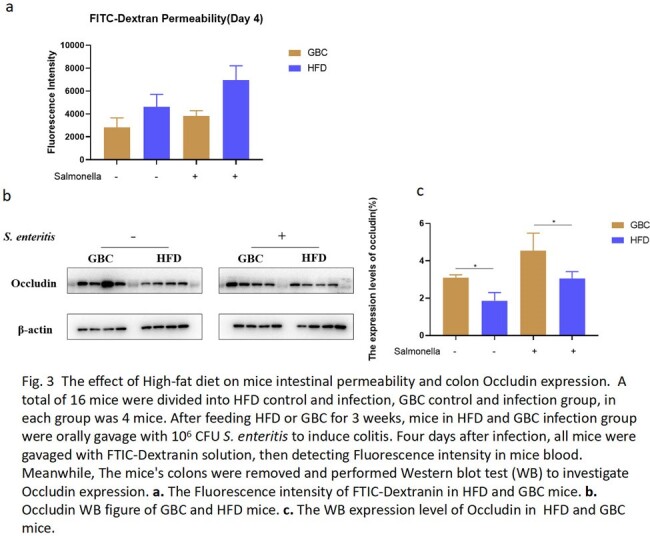

**Conclusion:**

Our findings reveal a significant increase in Salmonella infection-induced mortality associated with HFD. HFD increased host susceptibility to Salmonella infection maybe due to HFD increasing intestinal permeability and altering gut microbiota.

**Figure:**
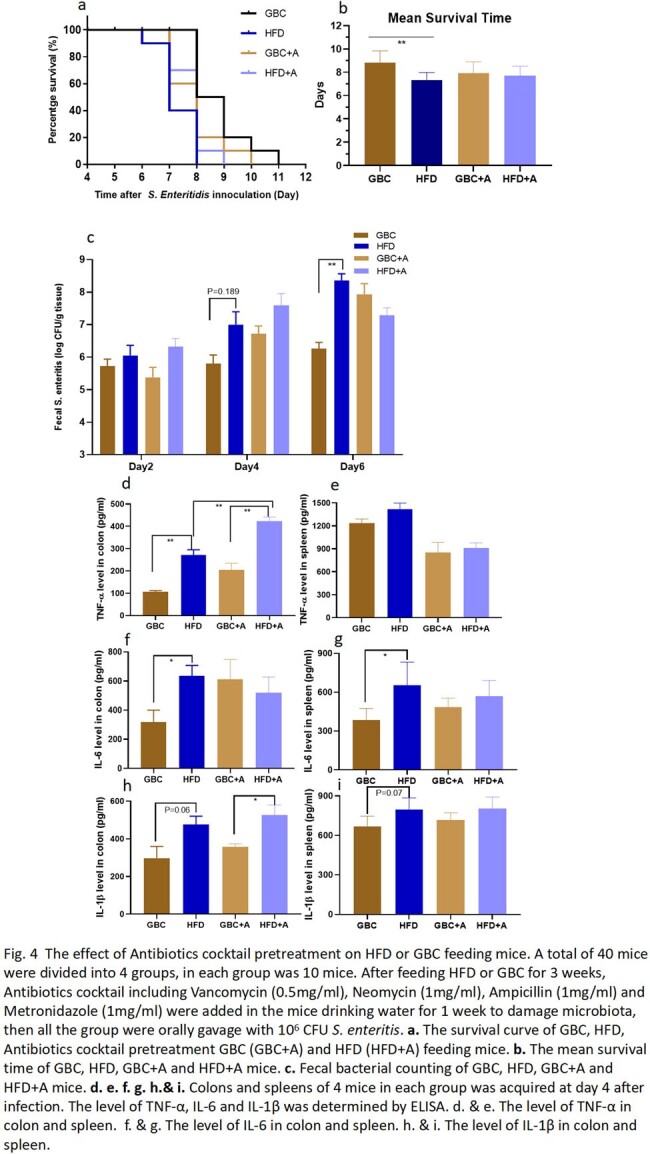

**Disclosures:**

**All Authors**: No reported disclosures

